# Counting the Founders: The Matrilineal Genetic Ancestry of the Jewish Diaspora

**DOI:** 10.1371/journal.pone.0002062

**Published:** 2008-04-30

**Authors:** Doron M. Behar, Ene Metspalu, Toomas Kivisild, Saharon Rosset, Shay Tzur, Yarin Hadid, Guennady Yudkovsky, Dror Rosengarten, Luisa Pereira, Antonio Amorim, Ildus Kutuev, David Gurwitz, Batsheva Bonne-Tamir, Richard Villems, Karl Skorecki

**Affiliations:** 1 Molecular Medicine Laboratory, Rambam Health Care Campus, Haifa, Israel; 2 Department of Evolutionary Biology, University of Tartu and Estonian Biocentre, Tartu, Estonia; 3 Leverhulme Centre for Human Evolutionary Studies, University of Cambridge, Cambridge, United Kingdom; 4 Data Analytics Research Group, IBM T.J. Watson Research Center, Yorktown Heights., New York, United States of America; 5 Rappaport Faculty of Medicine and Research Institute, Technion, Israel Institute of Technology, Haifa, Israel; 6 Instituto de Patologia e Imunologia Molecular da Universidade do Porto (IPATIMUP), Porto, Portugal; 7 Faculdade de Medicina, Universidade do Porto, Porto, Portugal; 8 Faculdade de Ciências, Universidade do Porto, Porto, Portugal; 9 Institute of Biochemistry and Genetics, Ufa Research Center, Russian Academy of Sciences, Ufa, Russia; 10 Department of Human Genetics, Sackler School of Medicine, Ramat Aviv, Israel; University of Glasgow, United Kingdom

## Abstract

The history of the Jewish Diaspora dates back to the Assyrian and Babylonian conquests in the Levant, followed by complex demographic and migratory trajectories over the ensuing millennia which pose a serious challenge to unraveling population genetic patterns. Here we ask whether phylogenetic analysis, based on highly resolved mitochondrial DNA (mtDNA) phylogenies can discern among maternal ancestries of the Diaspora. Accordingly, 1,142 samples from 14 different non-Ashkenazi Jewish communities were analyzed. A list of complete mtDNA sequences was established for all variants present at high frequency in the communities studied, along with high-resolution genotyping of all samples. Unlike the previously reported pattern observed among Ashkenazi Jews, the numerically major portion of the non-Ashkenazi Jews, currently estimated at 5 million people and comprised of the Moroccan, Iraqi, Iranian and Iberian Exile Jewish communities showed no evidence for a narrow founder effect, which did however characterize the smaller and more remote Belmonte, Indian and the two Caucasus communities. The Indian and Ethiopian Jewish sample sets suggested local female introgression, while mtDNAs in all other communities studied belong to a well-characterized West Eurasian pool of maternal lineages. Absence of sub-Saharan African mtDNA lineages among the North African Jewish communities suggests negligible or low level of admixture with females of the host populations among whom the African haplogroup (Hg) L0-L3 sub-clades variants are common. In contrast, the North African and Iberian Exile Jewish communities show influence of putative Iberian admixture as documented by mtDNA Hg HV0 variants. These findings highlight striking differences in the demographic history of the widespread Jewish Diaspora.

## Introduction

Contemporary Jews, whose number is estimated at 13 million [1], can be divided to Ashkenazi and non-Ashkenazi, which are each in turn comprised of numerous different constituent communities. Ashkenazi refers to Jews whose recent ancestry over the past millennium traces to Central and Eastern Europe. The geographically much more widespread non-Ashkenazi Jewish communities are also culturally more diverse, and are comprised of the Jewish communities that have continuously resided in the Near and Middle East and in North Africa and in different geographic locations to which Jews fled or to which they were deported including the Iberian expulsion in 1492–1495. These communities also share similar religious rituals, probably due to their presumed common historical origin from the descendants of the much earlier Babylonian exile. As a result of common ritual practices, they are sometimes collectively referred to as the Sephardic (Spanish) or Mizrahi (Eastern) Jews. However, the term Sephardic might better be reserved to designate those Jewish communities that emanated directly following the Iberian expulsion. Moreover, neither the term Sephardic nor Mizrahi takes fully into account some additional Jewish communities such as the Italian and Yemenite. Therefore, the term non-Ashkenazi Jews, cumbersome as it is, encompasses here all non-Ashkenazi communities, currently estimated to comprise about 5 million individuals [1]. While the genetic ancestry of the Ashkenazi has been investigated recently in some depth in terms of both male and female lineages, by means of the male-specific portion of the Y chromosome and the mtDNA respectively [2]–[5], the comparative data currently available on the non-Ashkenazi Jews is scant [6], [7]. These studies suggested geographically independent founding of the different Jewish communities. It is now possible to address the question of the matrilineal origin of these communities using phylogenetic resolution at maximum depth, and also to extend phylogeographic comparisons with a much wider range of reference populations.

We have recently made use of a combined phylogenetic/phylogeographic strategy that includes complete mtDNA sequence analysis in order to portray founding events in matrilineal descent [5]. This strategy was applied to the Ashkenazi and uncovered a limited number of founding maternal lineages which account for nearly a half of the contemporary Ashkenazi Jewry. Accordingly, we applied these same principles to “count the founding mothers”, and where possible date and localize their origin, for the more complex case study of the non-Ashkenazi Jewish communities, each of which was subject to different and changing demographic influences during the course of their respective Diaspora histories. In each community, we identified the contemporary most frequent founding lineages that collectively encompass at least 40% of their respective mtDNA gene pool. To estimate the approximate coalescence ages of each of the identified frequent founding lineages, new analytical tools were developed.

## Results

Of the 1142 non-Ashkenazi Jewish mtDNA genomes (Table 1), 1069 belonged to communities represented by more than 25 samples and were chosen for further analysis of their founding lineages. The fourteen non-Ashkenazi Jewish communities fulfilling this criterion were from Azerbaijan, Mountain Jews (58); Georgia (74); Ethiopia, Beta Israel (29); India: a) *Mumbai,* B'nei Israel (34), b) Cochin (45); Iran (82); Iraq (135); Libya (83); Morocco (149); Tunisia (37); Portugal (Belmonte, 30); Bulgaria (71); Turkey (123); and Yemen (119). All samples were collected in Israel. Hg frequencies (incl. 95% confidence intervals) for each of the communities, based on the analysis of coding region positions and appropriate control region motifs (Table S2), are presented in Table S3.

**Table 1:** List of populations and communities included in this study

Population/Community	N
NON-ASHKENAZI JEWS	1142
*Caucasian*	132
Azerbaijan (Mountain Jews)	58
Georgia	74
*Ethiopian* (Beta Israel)	29
*Indian*	79
Bombay (B'nei Israel)	34
Cochin	45
*Near and Middle Eastern*	251
Afghanistan	1
Iran	82
Iraq	135
Kurdistan, Iraq	12
Uzbekistan	17
Syria	4
*North African*	289
Algeria	20
Libya	83
Morocco	149
Tunisia	37
*Iberian Exiles* a	243
Portugal (Belmonte)	30
Bulgaria	71
Italy	9
Turkey	123
Former Yugoslavia	1
Miscellaneous	9
*Yemenite*	119
NON-JEWISH	253
Bedouins	58
Cherkess, Israel	8
Druze	77
Palestinians	110
**Total**	1395

a The designation “Iberian Exiles” is applied to those Jewish communities which emanated from the Iberian Peninsula after the exile of Jews in 1492.

### Control region vs. complete mtDNA sequence comparisons

**Figure 1:**
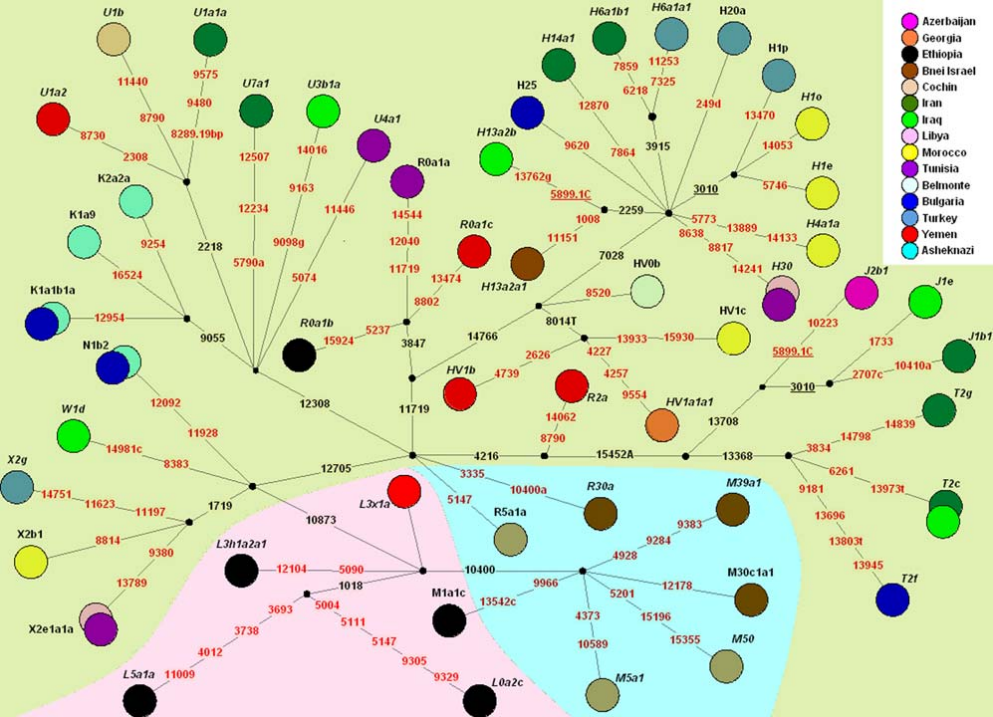
A median joining network representing all founding lineages found among Jewish communities and comprised of the 49 novel complete mtDNA sequences from non-Ashkenazi Jews and the four Ashkenazi lineages previously reported by Behar et al. [5]. The nodes represent the Jewish communities in which the lineages were found. The network should not be regarded as demonstrating all nested bifurcations known to exist under each Hg, but as a schematic tree indicating the wide range of Jewish lineages spread throughout the human mtDNA phylogeny. Nucleotide positions in red correspond to the diagnostic positions inferred from the complete sequences information and checked in all samples suspected as a monophyletic lineage from the control region information. The three complete sequences that could not be clearly proved as monophyletic lineages are marked by an asterisk. Mutations are transitions, unless the base change is explicitly indicated. Deletions are indicated by a “d” following the deleted nucleotide position. Insertions are indicated by a dot followed by number and type of inserted nucleotide(s). The background colorization denotes the gross affiliation of the lineages into the following geographic mtDNA gene pools: green for West Eurasia, pink for Africa and blue for South Asia/ India. The information of the reported samples is presented in Table 2. To create the topology map we have applied the reduced median algorithm (*r* = 2), followed by the median-joining algorithm (*epsilon* = 2) as described at the Fluxus Engineering Website (www.fluxus-engineering.com).

Table S1 presents genotyping results for all samples. A total of 49 putative founding lineages were identified and further analyzed as outlined above. Once again, we put forward herein and below the narrow designation for “a founder lineage” as being present in the sample of the contemporary community at a frequency equal to or greater than 5%, based upon the complete mtDNA genomic sequence for that lineage. Figure 1 illustrates the overall diversity of the non-Ashkenazi (this study) and Ashkenazi [5] Jewish founding lineages and their distribution within the various Jewish communities. One X2b Moroccan Jewish putative founding lineage was analyzed using 2 complete mtDNA sequences. One putative founding lineage in Hg T2 was shared by Iraqi and Iranian Jews, and was assessed by two complete mtDNA sequences. Two putative founding lineages (one in Hg H and one in Hg X2e) were shared by Libyan and Tunisian Jews, and were assessed by the same complete mtDNA information. Therefore, the current study yielded a total of 49 novel complete mtDNAs. The detailed phylogenetic tree drawn from the complete mtDNA information is shown in Figure S1.

**Figure 2:**
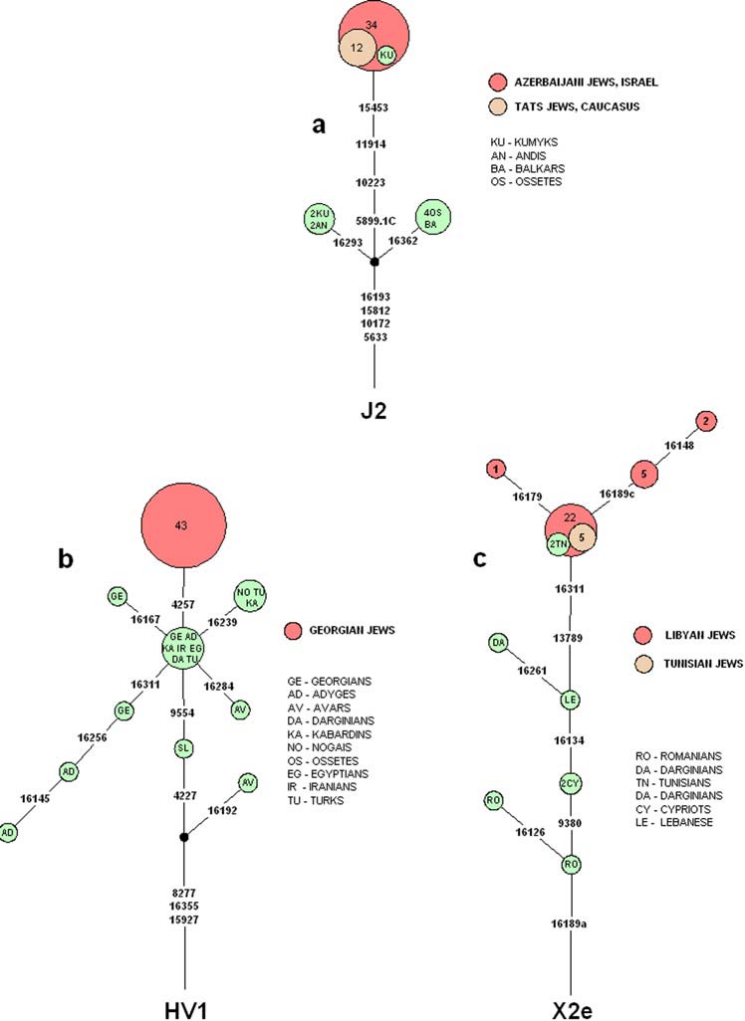
Phylogeny networks of the Azerbaijani (a), Georgian (b) and Libyan (c) Jewish case studies. The trees encompasses the coding region diagnostic positions (in bold) derived from the complete sequence information and the HVS-I information of all samples included. The same considerations detailed in figure 1 are relevant here. Circle sizes are proportional to the haplotype frequency in the sample.

For 37 of the 49 putative founding lineages assessed, diagnostic coding region single nucleotide polymorphisms (SNP) (described in Methods) have been identified (Table S4). The identifications of such diagnostic coding region SNPs implicate the corresponding samples as belonging to one founding lineage. Often, the inferences obtained from the complete mtDNA sequence paralleled the information from the control region. For example, in Georgian Jews, the presence of the control region haplotype 16067-16355-150-263 suggested the presence of a monophyletic clade *HV1a1a1* (the use of italic fonts in a clade name is explained in Material and Methods) within Hg HV1–an assumption that was confirmed by genotyping three private coding region positions 4227, 4257 and 9554 (Figure 2b), discerned from the complete mtDNA sequences (Table S1). One additional Georgian Jewish sample belonged to Hg HV1, but did not share the control region substitutions at 16355 and 150, and also lacked the coding region transitions specific for the *HV1a1a1* founder. To the contrary, two mtDNA genomes found among Azerbaijani Jews exactly matched the Georgian founding lineage and indeed were shown to share the identical coding region position variants as well.

The remaining 12 putative founding lineages showed a variety of relationships between the control region and complete sequence information within the hierarchy of coding region SNPs (Table S4). For example, in Cochin Jews, 12 samples were ascertained as belonging to Hg *M5a1* (Table S1, Figure S1), of which all were considered to belong to a monophyletic clade, as they all shared the first hypervariable segment (HVS-I) haplotype 16223-16257-16519-73-263. However, the two putative diagnostic coding region positions that were examined (4373 and 10589), clustered these samples into two nested groups. All samples shared position 4373, but only 11 of the 12 samples shared position 10589, the remaining one representing the likely ancestral haplotype or a sister lineage within the sub-clade. The Iraqi Jewish mtDNAs within Hg J1 had the hallmark of control region haplotype 16069-16126-16145-16222-16261-73-263-295 and thus was initially considered to belong to Hg J1b. Following complete sequence analysis, it became apparent that the lineage does not share positions 5460 and 13879 with Hg J1b and therefore represents a split on the link from J1 to J1b labeled herein as J1b'e. Two mutations (1733 and 8269), were examined in all 14 samples with the relevant control region motif (Tables S1 and Table S4). Position 8269 was shared by all of them, suggesting their descent from the same deep J1 branch, while position 1733 turned out not to be in the derived state in 6 samples, but rather all contained the gain of a substitution at position 152, in the second hypervariable segment (HVS-II). Hence, the J1 mtDNA lineages in Iraqi Jews descend from two rather than one founding mothers with a yet not fully resolved location under J1b'e and *J1e* (Figure S1). The same pattern repeated itself in Moroccan Jewish Hg *H4a1a*, Libyan-Tunisian Jewish Hg *H30* and Yemenite Jewish Hg *R0a1c*, putative founding lineages that were also shown to either be two daughter or two sister lineages within the sub-clade (Tables S1 and Table S4). Yet in other cases, mtDNAs with identical control region sequences within a community, generally indicative of very recent common ancestry, showed a rather high level of heterogeneity at their coding regions, consistent with a considerably more remote shared ancestry. Among Moroccan Jews, all 8 samples designated as Hg X2b1 (Table S1 and Table S4, Figure S1) shared the identical control region haplotype 16183C-16189-16223-16248-16278-16519-73-153-195-225-226-263. The first randomly chosen complete mtDNA sequence determined among them revealed three substitutions (at nps 1555, 2308 and 8814) which were not present among previously published X2 sequences [8]. Variation at nps 1555 and 2308 was assayed among the rest of Moroccan Jewish X2b1 sequences, and none of these samples were found to show the derived state at these positions. A second randomly chosen sample was also fully sequenced, and showed a derived allele at position 8814 as in the previous sample, plus two additional mutations at positions 6335 and 8277. Further genotyping revealed that this single informative variant at position 8814 was shared among all samples, confirming their remote common ancestry within X2b1. An analogous scenario was observed in the Bulgarian Jewish Hg *T2f* lineage (Tables S1 and Table S4).

In four putative founding lineages identified among Moroccan (*H1e*, *H4a1a*), Bulgarian (H25) and Turkish (H1p) Jews, the number of samples actually found to belong to one monophyletic clade was substantially lower than the suspected number, based solely on the initial inspection of their control region variation (Table S4). Their control region haplotypes contain positions 16519C and 263G (Table S4). We found, however, that even within regional communities, these mutations provide no phylogenetically informative value within Hg H. Finally, in the Hg X2e1a1a Libyan-Tunisian Jewish putative founding lineage, no coding region hierarchy was observed, but the HVS-I based tree clearly deviated from a star phylogeny and suggested the expansion of several sister clades (Table S1 and Figure 2c).

### Coalescence analysis

Table 2 provides the coalescence analysis, with 90% confidence intervals calculated differently for lineages with starlike and non-starlike phylogeny, as described in Methods. In some lineages, such as the Azerbaijani and Georgian Jewish *J2b1* and *HV1a1a1*, the coalescence age and its confidence intervals fall well within 2,000 years. In most others, such as the Iranian Jewish *H14a1* lineage, the coalescence age but not the confidence intervals fall within this time frame. In many cases limited sample sizes generate wide confidence intervals, despite of the lack of variation. Yet in other lineages, such as the Bulgarian Jewish H25 lineage, the estimated coalescence age is itself already outside the historical time frame of 2,000 years.

**Table 2:** A list of complete mtDNA based lineages established for all frequently present variants in the Jewish communities studied

Community (N)	Estimated size of the community^e^	Lineage definition by complete sequence	% in the community	control region haplotype (16024-300)	Phylogeny^c^	Coalescence (bounds) (y)
Azerbaijan (58)	100,000	*J2b1*	58.6%	16069 16126 16193 73 150 152 263 295	Starlike	484 (133–1766)
Georgia (74)	100,000	*HV1a1a1*	58.1%	16067 16355 150 263	Starlike	580 (197–1706)
Ethiopia, Beta Israel (29)	120,000	*R0a1b*	10.3%	16126 16305T 16362 58 60.1T 64 263	Starlike	0 (0–7091)
		*L3h1a2a1*	6.9%	16148 16192 16223 16234 16311 16399 73 146 152 263	Starlike	0 (0–9016)
		*L5a1a*	6.9%	16129 16148 16166 16180d 16187 16189 16223 16278 16311 16355 16362 73 152 182 195 247 263	Starlike	0 (0–9016)
		M1a1c	6.9%	16093 16129 16189 16223 16249 16311 16359 16519 73 195 263	Starlike	0 (0–9016)
		*L0a2c* d	10.3%	16148 16172 16187 16188A 16189 16214 16223 16230 16234 16311 16519 64 93 95C 152 189 236 247 263	Starlike	5537 (1883–16282)
India, B'nei Israel (34)	65,000	*M39a1*	41.2%	16166 16223 16311 16519 56d 58A 65.1T 73 152 207 263	Starlike	2755 (857–5668)
		M30c1a1	11.8%	16069 16162d 16223 16519 73 146 195A 263	Starlike	0 (0–5843)
		*R30a*	8.8%	16292 497 16519 73 263	Starlike	0 (0–7091)
		*H13a2a1*	5.9%	16519 183 263	Starlike	0 (0–9016)
India, Cochin (45)	10,000	*M5a1* d	26.7%	16223 16257 16519 73 263	Starlike	632 (111–3578)
		*M50*	17.8%	16223 16263 16519 16527 73 152 263	Starlike	1785 (490–6510)
		R5a1a	11.1%	16266 16304 16311 16519 16524 73 93 200 263	Starlike	0 (0–4968)
		*U1b*	11.1%	16093 16129 16189 16222 16249 73 152 263 285	Starlike	0 (0–4968)
Iran (82)	>150,000	*H6a1b1*	11.0%	16284 16362 16482 16519 239 263	Starlike	809 (143–4583)
		*H14a1*	8.5%	16256 16352 146 263	Starlike	0 (0–3824)
		*T2g*	6.1%	16126 16148 16294 16296 16519 73 200 263	Starlike	0 (0–4968)
		*U1a1a* d	6.1%	16183C 16189 16249 73 246 263 285	Starlike	1293 (228–7327)
		*J1b1*	4.9%	16069 16145 16261 16290 16519 73 150 263 271 295	Starlike	0 (0–5843)
		*T2c1* e	4.9%	16126 16292 16294 16296 16519 73 152 263	Starlike	291 (51–1649)
		U7a1^d^	6.1%	16318T 16519 73 151 152 263	Starlike	5173 (2012–13303)
Iraq (135)	>250,000	*T2c1* e	17.0%	16126 16292 16294 16296 16519 73 152 263	Starlike	291 (51–1649)
		J1b'e^f^	10.4%	16069 16126 16145 16222 16261 73 263 295	non-Starlike	1102 (0–2384)
		J1b'e(x*J1e*)^f^	5.9%	16069 16126 16145 16222 16261 73 263 295	Starlike	0 (0–3429)
		*J1e* f	4.4%	16069 16126 16145 16222 16261 73 263 295	Starlike	0 (0–4322)
		*U3b1a*	8.1%	16086 16343 73 150 263	Starlike	681 (120–3860)
		*H13a2b*	7.4%	16311 16519 263	Starlike	2219 (406–5395)
		*W1d*	7.4%	16223 16260 16298 16519 73 194 195 200 204 207 263	Starlike	0 (0–2842)
Libya (83)	>40,000	X2e1a1a^g^	39.8%	16134 16189A 16223 16278 16311 16519 73 153 195 225 263	non-Starlike	4461 (0–9415)
		*H30* f,g	18.1%	16192 195 263	Starlike	0 (0–1990)
Morocco (149)	>250,000	*H1e*	6.7%	16519 263	non-Starlike	4439 (590–8287)
		*H4a1a* f	6.0%	195 263	non-Starlike	809 (0–2140)
		*H4a1a* f	3.4%	195 263	Starlike	0 (0–4968)
		*H4a1a* f	2.7%	195 263	Starlike	0 (0–5843)
		HV1c	4.7%	16067 16172 182 263	Starlike	0 (0–3824)
		*H1o*	1.3%	16519 263 267	Starlike	0 (0–9016)
		X2b1	5.4%	16183C 16189 16223 16248 16278 16519 73 153 195 225 226 263	Starlike	3042 (1183–7822)
Tunisia (37)	>100,000	X2e1a1a^g^	16.2%	16134 16189A 16223 16278 16311 16519 73 153 195 225 263	non-Starlike	2762 (315–5208)
		*H30* f,g	10.8%	16192 195 263	non-Starlike	3880 (0–8637)
		R0a1a	8.1%	16126 16362 58 60.1T 64 263	Starlike	0 (0–7091)
		*U4a1*	8.1%	16356 16519 73 195 263	Starlike	0 (0–7091)
Belmonte (30)	400	HV0b	93.3%	16298 72 195 198 263	Starlike	0 (0–1118)
Bulgaria (71)	>50,000	K1a1b1a	8.5%	16224 16234 16311 16519 73 114 263	non-Starlike	3976 (0–9366)
		H25	8.5%	16519 263	non-Starlike	3375 (0–7513)
		N1b2	5.6%	16145 16176A 16223 16390 16519 73 152 263	Starlike	651 (278–1524)
		*T2f* d	4.2%	16126 16153 16294 16519 41 73 150 263	Starlike	5537 (1883–16282)
Turkey (123)	>500,000	*X2g* d	5.7%	16183C 16189 16278 16519 73 195 225 263	Starlike	2986 (1016–8781)
		H20a	4.9%	16218 16328A 16362 249d 263 292	Starlike	2250 (617–8204)
		*H6a1a1*	4.1%	16311 16362 16482 239 263	Starlike	1293 (228–7327)
		H1p	2.4%	16519 263	Starlike	0 (0–7091)
Yemen (119)	>50,000	*R0a1c* f	11.8%	16126 16304 16362 58 60.1T 64 152 263	non-Starlike	1102 (0–2384)
		*R0a1c* f	8.4%	16126 16304 16362 58 60.1T 64 152 263	Starlike	632 (111–3578)
		*R0a1c* f	1.7%	16126 16304 16362 58 60.1T 64 152 263	Starlike	0 (0–9016)
		*R2a*	11.8%	16071 16188 16223 73 146 152 263	Starlike	0 (0–2117)
		*HV1b*	10.1%	16067 16274 263	Starlike	0 (0–2426)
		*L3x1a*	8.4%	16169 16223 16278 16519 73 150 189 199 204A 263	Starlike	2219 (406–5395)
		*U1a2*	5.0%	16162d 16182C 16183C 16189 16249 16311 73 152 263 285	Starlike	0 (0–4322)
Ashkenazi^f^ (583)	8,000,000	K1a1b1a	19.4%	16224 16234 16311 16519 73 114 263	non-Starlike	3976 (0–9366)
		K1a9	7.0%	16225 16234 16311 16519 73 114 263	Starlike	2211 (161–1393)
		K2a2	4.3%	16226 16234 16311 16519 73 114 263	Starlike	827 (281–2431)
		N1b2	9.6%	16145 16176A 16223 16390 16519 73 152 263	Starlike	651 (278–1524)

a The sign > is used in all communities in which estimates for the community size is available only upon moving from the Diaspora. Due to recent intra-Jewish admixture contemporary estimation of the number of people descending from the original total are not available.

b Hg names in italic fonts indicate them to be prospective candidates of clades to be defined properly in the future

c Stralike denote a phylogenetic cluster in which the derivatives are one step away from the central node.

d Coding region hierarchy was observed in these lineages

e Lineage was shared among Iraqi and Iranian Jews

f Coding region hierarchy was observed in these lineages and suggested two sub-clades. Coalescence analysis for the two sub-clades is shown.

g Lineage was shared among Libyan and Tunisian Jews

h The Ashkenazi data follows Behar et al. [5].

### The founding lineages

Based on the coding region analysis, we extended the initial number of 49 putative lineages (Table S4) to 53 potentially founding lineages, because the coding region analysis suggested that four putative founding lineages (J1b'e*/J1e*, *H4a1a*, *H30* and *R0a1c*) were each comprised of 2 daughter sub-lineages. For 52 of the 53 lineages, confidence intervals covered 2,000 years as a potential coalescence age, and are therefore concordant with the founder event occurring during the last 2,000 years. The only lineage for which the confidence interval did not cover 2,000 years is the Iranian Jewish *U7a1*. However, two additional lineages, *L0a2c* and *T2f*, gave a coalescence estimate larger than 4000 years, with their confidence intervals greatly exceeding 2,000 years. Thus, we can state that in general, while almost the entire set of founder lineages identified herein is consistent with the constraint of a coalescence age within the last 2,000 years, it is likely that some may have started to expand earlier. It should be recalled that the imposed 2,000 year constraint adds a degree of stringency–and relaxing this to values greater than 2,000 years would be expected to result in the inclusion of more lineages within the time from expansion.

The Jewish community of the Caucasus also known as Mountain Jews is believed to have been established during the 8^th^ century C.E. in the region corresponding to Dagestan and the current state of Azerbaijan as a result of a movement of Jews from Iran. Indeed, this community shows a striking maternal founding event, with 58.6% of their total mtDNA genetic variation tracing back to only one woman carrying an mtDNA lineage within Hg J2b. This lineage was chosen as one of our three exemplary case studies, presented below (Table 3, Figure 2a). The Georgian Jewish community, considered to have been established in the 6^th^ century C.E., similarly shows a founding event with 58.1% of its total mtDNA variation tracing back to one woman. This particular mtDNA lineage within Hg HV1 was chosen as an additional case study for further phylogeographic resolution (Table 4, Figure 2b).

**Table 3:** Case study: the mtDNA founder of the Azerbaijani Jews

Population/Community	HVS-I	N studied	5633C	5899	10223C	11914G	15453T
**Jews**							
Azerbaijan^a^	16069 16126 16193	34	T	.1C	T	A	C
Ashkenazi	16069 16126 16193 16278	1	T		C	G	T
	16069 16126 16193 16265T	1	T		C	G	T
	16069 16126 16193 16362	1	T		C	G	T
Tats^a^	16069 16126 16193	2	T	.1C	T	A	C
**Non-Jews**							
*Caucasus*							
Andis	16069 16126 16193 16293	2	T		C	G	T
Balkars	16069 16126 16193 16362	1	T		C	G	T
Kumyks	16069 16126 16193 16293	1	T		C	G	T
	16069 16126 16193	1	T	.1C	T	A	C
Ossetes	16069 16126 16193 16362	1	T		C	G	T
*Near and Middle East*							
Cyprus	16069 16126 16193	1	T		C	G	T
Egypt	16069 16126 16148 16193 16256 16274	1	T		C	G	T
Iran	16069 16126 16193 16274	1	T		C	G	T
Lebanon	16069 16126 16193 16270	1	T		C	G	T
Syria	16048 16069 16126 16193	1	T		C	G	T
Turkey	16069 16126 16193	2	T	.2C	C	G	C
*Europe*							
Albania	16051 16069 16126 16169 16193	1	T		C	G	T
Bosnia	16069 16126 16193 16195	1	T		C	G	T
Estonia	16069 16126 16168 16193 16278	1	T		C	G	T
Lithuania	16069 16126 16193 16278	1	T		C	G	T
	16069 16126 16193 16278	1	T		C	G	T
Moldavia	16069 16126 16193 16278	1	T		C	G	T
Moksha	16069 16126 16193 16278	1	T		C	G	T
Russians	16069 16126 16193 16278	1	T		C	G	T
	16048 16069 16126 16193	1	T		C	G	T
Italy, Siena	16069 16126 16148 16193 16259	1	T		C	G	T
Slovakia	16069 16126 16193 16278	1	T		C	G	T
	16069 16126 16193	1	T		C	G	T
Ukraine	16069 16126 16193 16278 16311	1	T		C	G	T

a These two sister populations are labeled as Azerbaijani Jews and Tats Jews if collected in Israel and the Caucasus, respectively.

**Table 4:** Case study: the mtDNA founder of the Georgian Jews

Population/Community	HVS-I	N studied	4227A	4257A	8277T	9554G	15927G^a^
**Jews**							
Georgia	16067 16355	43	G	G	C	A	A
	16067 16183C 16189	1	A	A	T	G	G
Yemen	16067 16274	12	A	A	T	G	G
	16067	5	A	A	C	G	G
Morocco	16067 16172	7	A	A	T	G	G
	16067 16311	3	A	A	T	G	G
**Non-Jews**							
*Caucasus*							
Georgia	16067 16167 16355	1	G	A	C	A	A
	16067 16311 16355	1	G	A	C	A	A
	16067 16291	1	A	A	T	G	G
	16067 16355	1	G	A	C	A	A
	16067 16092 16234	1	A	A	T	G	G
Adyges	16067 16145 16256 16311 16355	1	G	A	C	A	A
	16067 16256 16311 16355	1	G	A	C	A	A
	16067 16355	1	G	A	C	A	A
Avars	16067 16284 16355	1	G	A	C	A	A
Darginians	16067 16355	1	G	A	C	A	A
Kabardins	16067 16355	1	G	A	C	A	A
	16067 16239 16355	1	G	A	C	A	A
Nogays	16067 16239 16355	1	G	A	C	A	A
Ossetes	16067 16355	1	G	A	C	A	A
							
*Near and Middle East*							
Egypt	16067 16355	1	G	A	C	A	A
	16067 16356	1	A	A	T	G	G
Turkey	16067 16239 16355	1	G	A	C	A	A
	16067 16355	1	G	A	C	A	A
Iran	16067 16355	1	G	A	C	A	A
	16067 16192 16355	1	A	A	C	G	A
*Europe*							
Slovak	16067	1	A	A	T	G	G
	16067 16355	1	G	A	C	G	A

a Position 15927 was checked in the Jewish samples in only one sample from each community.

Multiple theories exist regarding the establishment of the Ethiopian Beta Israel community, of which the most widely cited posits a migration event of Hebrews to Ethiopia in biblical times. Because of small sample size, even doubletons could meet the >5% threshold for inclusion as a putative founding lineage and therefore the results should be interpreted with caution. Nevertheless, it was not possible to cumulatively account for 40% of the genetic mtDNA variation with the lineages ascertained, using the criteria applied in this study. The four most frequent lineages belonged to Hgs *R0a1b*, *L3h1a2a1*, *L5a1a* and M1a1c (Table 2) all frequent in the region [10] suggesting East Africa and not the Levant as their likely geographic origin.

The Indian Jewish community of Mumbai (known as B'nei Israel) oral history claim to have descended from Jews who reached the shores of India in the 2^nd^ century C.E. MtDNA analysis for this community shows a strong maternal founding event, with 41.2% of its total mtDNA genetic variation tracing back to one woman and 67.6% tracking back to four women (Table 2). The Indian Jewish community of Cochin myth claims the community to have emanated in the times of King Solomon and has had no documented contact with the B'nei Israel of Mumbai. This community also shows a strong maternal founding event, with 44.4% of its total mtDNA genetic variation tracing back to two women (Table 2). In both Indian Jewish communities, their mtDNA gene pool is dominated by Hg M sub-branches specific for the subcontinent [11], and therefore appears to be of clearly local origin. It is important to note that in agreement with an oral tradition of the two independent founding events for the respective communities, the prevailing sub-branches among B'nei Israel Hg M samples belong to Hgs *M39a1* and M30c1a1, while the Cochin Hg M sub-branches belong to Hgs *M5a1* and *M50* (Table 2).

The Jewish communities of Iraq and Iran constitute the oldest non-Ashkenazi Jewish communities outside the Levant and were established during the 6^th^ century B.C.E. For the Iranian (Persian) Jewish community sample set, we found that 41.5% of the mtDNA variation can be attributed to 6 women carrying mtDNA genomes that belong to sub-branches of Hgs *H6a1b1*, *H14a1*, *T2g*, *T2c1*, *U1a1a*, and *J1b1* (Table 2), all known to be present in West Eurasia. In this regard, it is noteworthy that though Hg H is the dominant European mtDNA Hg (40-50%), its sub-Hgs H6 and H14 are largely restricted to the Near East and the South Caucasus [12]. Similarly, we found that about 43% of the Iraqi Jewish community can be traced back to 5 women whose mtDNA belongs to Hgs *T2c1*, J1b'e/*J1e*, *U3b1a*, *H13a2b* and *W1d* (Table 2), all frequent in the Near and Middle East. Again, Hg H13 is typically the Near Eastern, not European variant of Hg H [12]. Consistent with our findings, an independent sample of Iraqi Jews reported in a previous study [7], contained eleven out of 20 individuals who carry mtDNA variants, that can be assigned to the five founding lineages identified in the current study.

The presence of Jews in North Africa spans from the Roman domination of this region, through the period of the Arab caliphate of Baghdad and finally to the arrival of Jews, exiled from Spain and Portugal at the end of 15^th^ Century. The Libyan and Tunisian Jewish communities share, as their two most frequent mtDNA variants, lineages in Hgs X2e1a1a and *H30* (Table S4). It is important to note that the Hg *H30* is split by the coding region information into 2 sub-lineages, one restricted to Libyan Jews and one primarily to Tunisian Jews. The maternal founding event in Libyan Jews is evident, as 39.8% of their mtDNAs could be related to one woman carrying the X2e1a1a lineage, supported by an earlier observation, where ten out of twenty Libyan Jews were found to share this haplotype [7]. A well pronounced, though less narrow founder event characterizes Tunisian Jewry, where 4 maternal lineages (Hgs X2e1a1a, *H30*, R0a1a and *U4a1*) contributed to 43.2% of the entire mtDNA variation. The shared Libyan-Tunisian X2e1 was chosen as the third case study for further phylogeographic resolution (Table 5, Figure 2c). The Moroccan Jewish community, known to be the largest of the three showed no evidence for a significant maternal founding event in the sense defined in the current study. The most frequent lineages, each accounted for no more than 6.0% of the entire mtDNA genetic variation, and only 12.7% of overall mtDNA variation could be explained by lineages with frequencies greater than 5%.

**Table 5:** Case study: The mtDNA founder of the Libyan Jews

Population/Community	HVS-I	N studied	9380G	13789T
**Jews**				
Libya	16134 16189A 16223 16278 16311	22	A	C
	16134 16179 16189A 16223 16278 16311	4	A	C
	16134 16189 16223 16278 16311	5	A	C
	16134 16148 16189 16223 16278 16311	2	A	C
Tunisia	16134 16189A 16223 16278 16311	6	A	C
**Non-Jews**				
Tunisia	16134 16189A 16223 16278 16311	2	A	C
Lebanon	16134 16189A 16223 16278	1	A	T
Darginians	16134 16189A 16223 16261 16278	1	A	T
Cyprus	16189A 16223 16278	2	A	T
Romania	16189A 16223 16278	1	G	T
	16126 16189A 16223 16278	1	G	T

It is worth stressing here that all predominant mtDNA lineages found among the North African Jewish communities belong to the general West Eurasian pool. Jews expelled from Spain and Portugal joined with those existing elsewhere or established new communities in many locations. At some locations, such as Bulgaria and Turkey, the influx was large enough to consider the entire community as a representative subset of the parental Jewish population in Spain. Our data does not support a narrow founding event in the establishment of the Bulgarian or Turkish Jewish communities. Two of the most prevalent mtDNA lineages in Bulgarian Jews were identical to those found among Ashkenazi Jews. It is also worth noting that both communities had a high prevalence of Hg H mtDNA genomes, which, while frequent in the Near East, has still a significantly higher prevalence in the Iberian Peninsula. The Iberian Jewish community of Belmonte, Portugal, listed under the Iberian exile communities, is comprised of only 300-400 people. The community survived for hundreds of years by adhering to a crypto-Judaic lifestyle. It is impossible to ascertain that the sampling within this community avoided putative recent maternal introgression events. A total of 93.3% of the mtDNA genomes in the Belmonte samples could be attributed to one mother, carrying an mtDNA lineage within Hg HV0b, and thereby likely narrowing down the ancestry of these crypto Jewish “communities” to one endogamously expanding family, at least on the maternal side.

The Yemenite Jewish community is thought to have been established in the second century CE. Here we found that 42.0% of the mtDNA variation in this community can be attributed to 5 women carrying mtDNAs that belong to sub-branches of Hgs *R0a1c*, *R2a*, *HV1b*, *L3x1a* and *U1a2*. While these Hgs, except *L3x1a*, can be considered as a part of the general West Asian mtDNA genetic pool, they have higher frequencies in East Africa and Yemen [10].

### Case studies

To illustrate a straightforward approach for a deeper phylogeographic resolution of a founding lineage we choose three case studies comprised of dominant mtDNA haplotypes among Azerbaijani, Georgian and Libyan-Tunisian Jews.

1. The Azerbaijani Jewish community is dominated by a J2b1 lineage (Table 2). We screened a selection of West Eurasian and North African population samples (N = 6076) for Hg J2 genomes that contained HVS-I motif 16069, 16126, 16193. Significantly, this large mtDNA selection contains a duplicate of the Azerbaijani Jewish community collected in Israel since the same community was also sampled directly in its Caucasus Diaspora homeland. This community is known in the Caucasus as Jewish Tats. Table 3 details the genotyping of 65 geographically and ethnically diverse J2b mtDNAs for private coding region mutations, identified by complete sequencing of the Azerbaijani Jewish sample. The positions examined included an indel at 5899 and transitions at 10223, 10914 and 15453. The reconstructed Azerbaijani Jewish J2b founder phylogeny is shown in Figure 2a. The Jewish Tat population showed identical findings as those observed among in its Israeli sister community as evidenced by the fact that the same J2b1 lineage was found in 12 out of the 23 Jewish Tats mtDNA genomes. The same mtDNA lineage was also found in one out of 111 Kumyk samples.

2. The Georgian Jewish mtDNA pool was dominated by the Hg *HV1a1a1* lineage. We screened our population samples for Hg HV1 variants that contained the same HVS-I mutation 16355 observed in Georgian Jews. We then chose a selection of geographically wide-spread HV1 samples containing a transition at position 16355 and a few samples that did not, and genotyped them for the three coding region mutations 4227, 4257 and 9554, identified in complete sequencing of the particular Georgian Jewish mtDNA founder lineage. Table 4 details the genotyping information of the samples included, and Figure 2b shows phylogenetic reconstruction of the Georgian Jewish HV1 founder. A substitution at position 4257 was restricted to Georgian Jews whilst all other mutations were shared by almost all other samples studied here, carrying the transition at 16355. Interestingly, the substitution at 4227 was missing also in the Caucasus region, including Georgian HV1a1a samples (Table 4), suggesting that this particular mutation might have been arisen within the Georgian Jewish community.

3. The Libyan and Tunisian Jewish communities shared among them an X2e1a1a lineage as the most frequent. We examined the two Libyan-Tunisian Jewish lineage-specific coding region mutations 9380 and 13789 in relevant samples at hand (Table 5, Figure 2c). Position 13789 appears uninformative, while 9380 was shared among Hg X samples from the Near East and Africa, but not from Europe, suggesting Near Eastern/ North African origin of the particular founder lineage.

## Discussion

The non-Ashkenazi Jewish communities studied herein pose an analytic challenge to population geneticists. First, the communities are spread across a broad and diverse phylogeographic area with sometimes well understood regional pools of maternal lineages (e.g. India) [11], [13]-[15], sometimes with clear regional signals (e.g. Yemen, Ethiopia) [10] and in other cases in regions where the host populations still require more thorough examination (e.g. Libya). Therefore, the attempt to estimate the size of the founder effect in a large number of separate communities, each prone to different fluctuating demographic events during their histories, the latter often relying on oral narratives rather than archival or other better substantiated historical records, is intrinsically more complex than studying phylogeographically less complex cases, such as the Ashkenazi Jews [4], [5].

### The founding mothers

Haplogroup-wise, the diversity of the joint Ashkenazi and non-Ashkenazi pool of “frequent maternal founders” is considerable (Figure 1). Indeed, from the reference list of West Eurasian-specific Hgs [9], only a minor one-Hg I–is missing. U5, the most frequent branch of Hg U in Europe, is likewise absent. However, the two, as well as U2e, N1a, N1c, are present in all the sample sets of the Jewish mtDNA pool, analyzed in this study, albeit at low frequency (Table S1).

The Jewish communities revealed three different patterns of founder effects in their maternal heritage. Firstly, the Belmonte, Azerbaijani, Georgian, B'nei Israel and Libyan communities show a striking paucity of founding lineages (Table 2, Figure 1). In these communities, a single mother was sufficient to explain at least 40% of their present-day mtDNA variation. The Cochin and Tunisian Jewish communities show an attenuated pattern with two founding mothers explaining >30% of the variation.

The Bulgarian, Turkish, Moroccan and Ethiopian communities show a different pattern, with heterogeneity in the pattern observed among these communities. None of these show any evidence for a narrow founder effect or depletion of mtDNA variation attributable to drift (Table 2, Figure 1). Interestingly, the first three of these communities were established following the Spanish expulsion and/or received large influxes of individuals from the Iberian Peninsula and high variation presently observed, probably reflects high overall mtDNA diversity among Jews of Spanish descent. Likewise, the mtDNA pool of Ethiopian Jews reflects the rich maternal lineage variety of East Africa.

The third and intermediate pattern is observed in the Jewish communities from Iraq, Iran, and Yemen. Indeed, these communities are long-standing Diaspora communities that have historical records consistent with a founding event, but not a narrow one.

Our definition of a founding lineage is best regarded as an “operational” definition, in which we searched for lineages, which following our extensive genotyping at the level of complete mtDNA sequences, accounted for over 5% of the total mtDNA diversity and coalesced earlier than 2,000 years ago in any given non-Ashkenazi Jewish community. This definition differs from previous formal definitions for a “founder type” that mandated that the sequence type under consideration be carried from a source population to a derived population [9]. Therefore, our frequency based approach leaves a few questions open. First, what percentage of the initial founders does the contemporary surviving founding lineages represent? Second, were the contemporary frequent lineages part of the original founding lineages or were they introduced later from the surrounding host population? Third, based on the number of founding lineages (i.e. one or five) that cumulatively accounted for 40% of the contemporary maternal gene pool observed in any given community, is it possible to reach conclusions regarding the Jewish community which experienced the narrowest actual historic founder effect or the strongest bottleneck? It is actually not possible to address these questions with a high degree of confidence using contemporary genetic data alone, because any phylogeny inferred from extant genetic variation relates only to the surviving founders. Therefore, it is clear that the contemporary frequent lineages represent only the successful surviving lineages, while any lineage present in the current community diversity (Table S1) is possibly a founding lineage. In addition, one must consider that an unknown number of founder lineages might have been lost. However, our approach does allow us to suggest that no matter what the origin of the identified in this study founding lineages was, they likely were a part of the population founding lineages or were introduced shortly after the establishment of the community, as it is unlikely that their current high frequency is the result of recent gene flow.

Finally, two circumstances could have camouflaged narrower founder effects: the community at its establishment was already large enough to avoid significant loss of diversity through drift. Alternatively, initial founders could have been limited, but have gained, during the community's evolution, an influx of different Jewish or other Semitic or non-Semitic groups that were adopted into the respective community. Nevertheless, consideration of the historical record of Ashkenazi Jews, their present-day fraction among world Jewry, contrasted with paucity of their founding lineages, seems to support the formulation that no matter whether the four dominant Ashkenazi founding lineages [5] are the result of a very narrow founder event, or a series of subsequent bottlenecks yielding extreme drift, their expansion from a small historical founding deme has been the most substantial among all Jewish communities.

### The phylogeographic quest

An important question relates to the geographic origin of the founding lineages, which can be the geographic region corresponding to the ancient Levant, the Diaspora region in which a particular community has resided during the relevant time frame, or a combination of both.

#### The location of the first Diaspora communities

The Iranian Jewish mtDNA is particularly rich in Hg H (30.5%, see Tables S1 and Table S3)–the variant of maternal lineages that constitutes on average more than 40% of the mtDNA variation in Europe. Hg H is also well represented in the Iraqi Jewish community with an overall frequency of 11.8% (Tables S1 and Table S3). Meanwhile, Hg H frequency in Ashkenazi Jews of recent European ancestry is 20.4% [4]. This raises an interesting question regarding the possible source of Hg H lineages among the various Jewish communities. Recent progress in the understanding of mtDNA variation in East and West Europe [16]-[18], as well as in the Near East [12] fits with the inference that at least three quarters of Iranian and Iraqi Jewish Hg H genomes belong to sub-Hgs H6, H13 and H14, characteristic of the Near Eastern–Central Asian variants of Hg H. In view of the historical records claiming the establishment of the North African Jewish communities from the Near Eastern Jewish communities, it is noteworthy that the communities do not share their respective major founding lineages.

#### Typically African mtDNA variants in non-Ashkenazi Jews

African-specific Hgs–variants of largely sub-Saharan Hg L(xM,N)-as well as more northern and eastern Hgs M1 and U6, do occur within the gene pools of some, though not all non-Ashkenazi Jewish communities (Table S3). Perhaps expectedly, such lineages have not been detected in the Caucasus area Jews (Azerbaijan, Georgia), nor among the Middle Eastern (Iranian), nor among Indian Jews (Cochin Jews, B'nei Israel). However, they were found in Ethiopian and Yemenite Jews (Tables S1 and Table S3), perhaps reflecting the mtDNA population structure of the host countries. In contrast, it is intriguing to find that the North African Jews (Moroccan, Tunisian, Libyan) possess only a very small fraction of Hg L(xM,N) lineages (2.2%) and, even more unexpectadly, seem to lack typically North African Hg M1 and U6 mtDNAs (Tables S1 and Table S3). In striking contrast, sub-Saharan L lineages are prevalent in North African Arab and Berber populations at frequencies around 20–25% (25.5% in Moroccans, 24.9% in Tunisians, 30.2% in Libyans; our unpublished data), yielding a difference exceeding an order of magnitude. Curiously, the Ashkenazi mtDNA pool of recent European descent includes Hg L(xM,N) at a frequency comparable to that among North African Jewry [4], [5]. Hence, the lack of U6 and M1 chromosomes among the North African Jews and the low frequency of Hg L(xM,N) lineages, renders the possibilty of significant admixture between the local Arab and Berber populations with Jews unlikely, consistent with social restrictions imposed by religious restrictions.

Therefore, one can observe among the West Eurasian–North African non-Ashkenazi Jewish Diaspora communities we have examined, three essentially different demographic scenarios. First the Middle East Jews, supposedly at least partially descendent from the earliest Assyrian (late 8th Century BCE) and Babylonian (6th Century BCE) Hebrew exiles, whose mtDNA pools virtually lack sub-Saharan L and North and East African-specific M1 and U6 mtDNA variants. Secondly, the Ashkenazi and North African Jews with a low, but still detectable share of L lineages with very low diversity. This low diversity is most easily explained by a limited number of unique Hg L(xM,N) founders. The third example brings together Ethiopian and Yemenite Jews, rich in Hg L(xM,N) and Hg M1 (in particular in Ethiopian Jews) (Tables S1 and Table S3). As far as Ethiopian and Yemenite Jews are concerned, the main observation here is not in the absolute frequency of Hg L(xM,N) among them, but rather its high diversity, in particular among Beta Israel (Tables S1 and Table S3). Furthermore, samples of Ethiopian and Yemenite Jewish mtDNA pools differ considerably in relative abundance of typically West Asian mtDNA lineages such as derivatives of HV1, JT and others (Tables S1 and Table S3), virtually absent in the former.

#### South Asian maternal ancestry in B'nei Israel and Cochin Jews

While the mtDNA pools in B'nei Israel (Gujarat province) and Cochin Jews (Kerala) in general resemble Indian-specific maternal lineage variation with its characteristic repertoire of regionally autochthonous mtDNA Hgs, such as Indian-specific M, R and N variants (Tables S1 and Table S3), more careful inspection reveals differences that even follow intra-peninsular variation. Thus, for example, Hg M5a2, the dominant mtDNA variant in Cochin Jews, is particularly frequent in Kerala [19]-[21], suggesting that a considerable fraction of Cochin Jewish maternal lineages are of local origin, as far as the huge peninsula is concerned. Yet it is interesting to note that the few Hg H mtDNAs, found in B'nei Israel mtDNA pool, belong to sub-Hgs *H13a2a1* and H14, which heretofore have not been reported in sampling elsewhere in Gujarat [13], but commonly found among Iranian and Iraqi Jews (Table S1and Table S3). Moreover, an exact HVR-I motif match of H14 haplotype among B'nei Israel is present in our limited sample of Italian Jews (Tables S1 and Table S3). Likewise, the Cochin Jews possess typically Western Eurasian U1 Hg mtDNAs, present in several non-Ashkenazi Jewish communities (Table 2). We have not noted U1a mtDNA in Indians of Kerala, but it has been reported to be present elsewhere in western India [13]. Accordingly, these findings provide indirect but suggestive evidence that the two Indian Jewish communities still encompass maternal lineages brought along by original founders from outside the local community.

Two possible explanations can be suggested for our general findings regarding Ethiopian and Indian Jews. Firstly, idea flow and not gene flow may have constituted the main basis for establishing these Jewish communities. It is possible that cultural and religious ideas were conveyed by a scant number of people that carried lineages that subsequently became largely extinct or that are too infrequent to be detected with the current sample sizes. Secondly, one may speculate that these Jewish communities were established by males who recruited local women in a number sufficient to make the community genetically sustainable without significant subsequent introgression.

#### Anatolian Jews and the Iberian Expulsion

The sojourn of Jews to Antolia during the last 2 millenia is particularly complex and one can assume that the variation in the Turkic Jewish mtDNA pool encompasses maternal lineages, reflecting different demographic events. They may derive from: (i) relatively recent (here–within the last few millennia) Levantine ancestry, “carried back” to Anatolia and elsewhere in the Near East by Sephardic (Iberian) Jews around 500 years ago; (ii) putative ancestral Iberian mtDNA variants, introduced into Sephardic gene pool by admixture during their long stay in this peninsula; (iii) Jewish mtDNA variants introduced to the Turkic Jewish mtDNA pool via different routes attrituable to the attractiveness of the centre during the periods of Byzantine and Ottoman dominance. (iv) Turkic Jewish mtDNA variants may encompass ancient Anatolian mtDNA variants, persisting there since much more remote prehistoric times (v) mtDNA variants of Romaniots Jews who lived in small numbers in the respective places to which the Spanish exiles fled in Anatolia. Unambigous differentiation between this plethora of demographic scenarios, extending over at least two millennia and further back, seems prohibitively daunting. However, phylogeography suggests at least some lines of reasoning. As a general remark, one can indicate that the Turkic Jewish mtDNA pool, although highly divergent, does not encompass mtDNA lineages, typical of East Asia with the exception of a rare example of N9 (Tables S1 and Table S3). Neither does it contain typically African mtDNA variants of the Hg L(xM,N) and Hg M1. A single Hg U6a1 mtDNA found (Tables S1 and Table S3) is not unexpected, because it is particularly frequent alongside the Mediterranean coast of Africa and can be found as well in Iberian Peninsula and in Italy [22].

Yet the most reliable hint in favour of the West Mediterranean “signature” among the Turkic Jews can be found by following Hg V. Although Hg R0 (formerly pre-HV) most likely originated in West Asia [23], [24], the phylogeography of Hg HV0 (formerly variants of pre-V and V) suggests its main expansion as having occurred following the last glacial maximum, from the Iberian Peninsula. HV0 is well represented throughout northwest Africa, but barely reaches the Near East [25]. Therefore, its distribution among Jewish Diaspora communities is of particular interest. Indeed, in the Belmonte Jews of Portugal, Hg HV0b nearly reaches fixation (Tables S1 and Table S3), suggesting a particularly selective founder event–the community has most probably arisen from one or very few families with a limited repertoire of maternal lineages. Consistent with the Iberian-NW African focus of Hg HV0, derivative lineages are clearly evident, albeit in small numbers, among different NW African Jewish communities–in contrast to their absence in the Caucasian (Azerbaijani, Georgian), Iraqi and Iranian Jewish Diasporas (Tables S1 and Table S3). Because different sub-Hgs of HV0 are present both in Iberia and NW Africa, it remains unclear whether the observed maternal lineages in these two communities reflect only Iberian introgression into the northwest African pool, or rather were introduced at the time of the establishment of the northwest African Jewish Diaspora. However, in the same context it is noteworthy that different variants of HV0 can be observed among Turkic Jews as well (Tables S1 and Table S3), in accord with historical records documenting the migration of a considerable fraction of Iberian Jewish exiles to Anatolia, including to Istanbul, directly after their expulsion from the Iberian peninsula. In the Anatolian Jewish community, as well as in other eastern Mediterranean destinations, including Jerusalem, the Iberian exiles may have even numerically dominated the original Jewish community. Therefore, the fact that the Turkic Jewish mtDNA pool has preserved different HV0 lineages in numbers, quite comparable to their presence among northwest African Jews (Tables S1 and Table S3), is an independent genetic signal of an admixture of Iberian Jewry with local Iberian populations, that may date back to the era of the Roman Empire or earlier.

#### The three case studies

While the foregoing inferences relate to particular communities, the understanding of the geographic origin of each of the founding lineages begs a more deeply layered phylogeographic investigation. The first case study uncovered similarities between the maternal ancestry of the Jewish Indo-Iranian speaking Tat population and the Israeli Azerbaijani Jewish (Mountain Jews, Juhuro, Dash Chufur) community, as is evident from the shared *J2b1* lineage that dominates in both mtDNA pools (Table 3, Figure 2a). This is not surprising as the two actually represent sister populations, sampled once in Israel and once in the Caucasus. Moreover, the two populations showed the same narrow founder effect, clearly supporting its existence already in the Diaspora. The finding of a single identical *J2b1* lineage among their Kumyk neighbors is likely the result of minor gene flow among the two populations. While the geographic origin of this lineage can certainly be attributed to West Eurasia or the Near East, its exact origin within this large region can not be pinpointed from our data.

The second example highlighted the Georgian Jewish *HV1a1a1* haplotype (Table 4, Figure 2b) and showed that it existed only in Georgian Jews. While it is clear that the ancestry of this lineage can be traced to the broad geographic swathe encompassing the Near and Middle East as well as the Caucasus region, even the level of resolution generated from the complete mtDNA analysis could not provide greater phylogeographic specificity, since equidistant ancestral lineages could be found in each of the three geographic locations.

The third case study addresses the shared Libyan-Tunisian X2e1a1a haplotype. Again, it became clear that the ancestry of this lineage can be similarly attributed to the broad geographic region encompassing the Near and Middle East and the Caucasus region (Table 5, Figure 2c), but unlike the Georgian case study, the particular haplotype was shared with non-Jewish Tunisians, encompassing 0.8% to the overall Tunisian mtDNA pool. In addition, no HVS-I variation was observed in non-Jewish Tunisians, while such variation was clearly observed in Jews, suggesting the possibility of gene flow into the host population from Jews.

### Comparison to prior scientific literature

Based on partial HVS-I sequence information, it has been reported earlier (Thomas et al. [6] that Jewish Diaspora communities were founded independently, and display low overall diversity. In some important aspects our results and conclusions differ significantly from those of Thomas et al. [6].

First, we have not found narrow founder effects as being an overall characteristic of the non-Ashkenazi Jewish communities. In fact, all of the numerically large Moroccan, Iraqi, Iranian and Iberian Exile non-Ashkenazi Jewish communities, show no evidence of such very narrow founder effects, whereas, and perhaps expectedly, only the smallest and most remote non-Ashkenazi Jewish communities are those with the fewest founding mtDNA lineages still in circulation. Next, Thomas et al. [6] highlighted the Ashkenazi community as exhibiting the least pronounced founder effect, while the non-Ashkenazi Jewish data set presented herein, actually accentuates by contrast the magnitude of the founder effect we have previously reported in Ashkenazi Jews [5].

Second, the approach taken by Thomas et al. [6] has known limitations. An illustration can be provided by the Moroccan Jewish community. Thomas et al. [6] found that 26% of the mtDNAs in this community share a modal HVS-I haplotype identical to the revised Cambridge Reference Sequence (rCRS) and therefore concluded that Moroccan Jews show one of the narrowest founder effects among all of the communities which they studied. While our study confirmed the observed high prevalence of rCRS haplotypes in the HVS-I region among Moroccan Jews, analysis at the complete sequence resolution revealed a strikingly different pattern with respect to the number of founder lineages (Table S1 and Table S4). Namely, three randomly chosen samples, sharing a HVS-I rCRS haplotype, were found to lie in three different branches of Hg H (Table 3), having thus only a very remote shared ancestry, dating back long before the Jewish Diasporas were established.

In summary, we have documented the most frequent maternal lineages among highly divergent non-Ashkenazi Jewish communities and characterized them at the level of complete mtDNA sequences. The phylogenetic approach taken in the current study of most non-Ashkenazi Jewish communities, coupled with a previous study on Ashkenazi Jews, reveals the mechanisms involved in the formation of the various extant patterns of mtDNA haplotype variation of the Jewish Diasporas, and taken together provides a nearly comprehensive picture of the maternal genetic landscape of the entire Jewish population. Some of the communities reveal strong founder effects, while in others an abundance of maternal lineages is evident. Mechanisms, such as recruitment of maternal lineages from host populations, including their occasional historic long-distance transfer to new settlements, have been likely operative. Taken together, these studies show that while the founding event for each community may have had an important role in shaping their current genetic structure, other factors related to migration and survival of founding lineages, are responsible for the assembled list of remnant lineages, stressing once again the importance of an interdisciplinary approach in the reconstruction of demographic histories of extant populations. Tracing, assembling, and counting a list of successful mtDNA founders, coupled with detailed phylogeographic knowledge, seems to be a promising avenue for the exploration of the genetic roots of the matrilineal ancestries of human populations and offers useful guidelines for the rapidly emerging quest for regionally resolved patterns in the genetics of common diseases.

## Materials and Methods

### Sampling

A total of 1395 samples from unrelated individuals were collected, of which 1142 were of non-Ashkenazi Jewish origin and 253 were of Near Eastern non-Jewish origin. The database was compared to the previously reported results for 583 Ashkenazi Jews. Table 1 details the populations studied, the communities comprising each population and the number of samples. All samples reported herein were derived from buccal swab or blood cell samples that were collected with informed consent according to protocols approved by the National Human Subjects Review Committee in Israel and Institutional Review boards of the participating research centers. Samples were recruited during scheduled public lectures in the field of archaeogenetics that addressed the general public, genealogical societies, heritage centers and the scientific community. In addition, the National Laboratory for the Genetics of Israeli Populations (http://www.tau.ac.il/medicine/NLGIP/) who concentrate on collecting and establishing human cell lines representing the various ethnic groups in Israel have donated samples to this study. Each of the subjects reported the birthplace of their mother, and maternal grandmother, and in many cases also of great grandmother.

### Nomenclature

Nucleotide positions 1-16569 refer to the position of the mutation in the revised Cambridge reference sequence [26]. Where labeling new clades (Hgs or sub-Hgs) in mtDNA phylogeny we follow the rules described in Richards et al. [27] and tried to keep our new labeling as accurate as possible. However, the wealth of information accumulating from complete mtDNA sequences is rapidly growing and ever burdening the mtDNA nomenclature. Therefore, to facilitate the track of our newly introduced names we refer to Table S5 which details for every founder haplotype a citation for the smallest enclosing clade that has so far been defined plus any new sub classification that was introduced in this study. In addition, we composed a phylogeny map of all previously reported clades of the human mtDNA phylogeny in which our founding lineages were found (Figure S2). While assembling the topology map we did not alter previous designations. We added new labeling for previously un-labeled bifurcations if they became important for our discussion. We use italic fonts (i.e. *T2f*) to label all haplotypes in which the position in the link to and from the haplotype of interest could not be fully appreciated from the availability of the complete sequence and the few coding region SNPs genotypes in the respective lineage. These designations and font usages are meant to facilitate reading, and are prospective candidates of clades to be fully defined in the future. Finally, some confusion in the literature is apparent in referring to Hg L. It is common practice to cumulatively refer to African clades as Hg L lineages. However, the term actually encompasses all sampled human mtDNAs including the Hgs representing the out of Africa exodus (L3)M and (L3)N. Therefore, we use the label Hg L(xM,N) in the text to refer to African L lineages other than M and N.

### Genotyping protocols

For all samples, sequences of the control region were determined from position 16024 to 00300, using the ABI Prism Dye Terminator cycle-sequencing protocols developed by Applied Biosystems (Perkin-Elmer), to provide an initial presumed Hg assignment for any given sample. The C-track length variation at positions 16182 and 16183 in HVS-I and the indels at positions 00309 and 00315 in HVS-II were excluded from further analyses (Table S1). Hg assignment was then confirmed, based on control and coding region Hg defining polymorphisms (Table S2) determined by means of restriction fragment length polymorphisms or direct sequencing.

Complete mtDNA sequences were obtained for 49 samples. For complete mtDNA sequencing, 18 primers were used to yield 9 overlapping fragments as previously reported [28]. After purification, the 9 fragments were sequenced by means of 56 internal primers to obtain the complete mtDNA genome [28]. Sequencing was performed on a 3730xl DNA Analyzer (Applied Biosystems), and the resulting sequences were analyzed with the SEQUENCHER software. The novel 49 complete mtDNA sequences reported herein have been submitted to GenBank (accession numbers EF556148–EF556196). Quality control was assured as follows: first, each base pair was determined once with a forward and once with a reverse primer; second, any ambiguous base call was tested by additional and independent PCR and sequencing reactions; third, all sequences were examined by two independent investigators.

### Analysis of putative founding lineages

Within a given community of interest mtDNA Hg identity shared between individuals does not necessarily indicate that they derive from a common ancestor that lived within the historical time frame of existence of the community. This is because more than one founder is likely to have existed within a given Hg, as we have shown using complete mtDNA sequencing for the Ashkenazi founding lineages within Hg K [5]. Therefore, in order to identify those founding mtDNA lineages that might have existed during the foundation of the community, we combined Hg identification with control region haplotype information as a first screening procedure to identify lineages of putative shared maternal ancestry, within the approximately 2,000 year time frame relevant to the history of the Jewish Diaspora. It is important to note that this is a conservative constraint since historical records clearly suggest the establishment of some of the Jewish communities (Iraq) to be 2,500 years ago and therefore the constraint could have been relaxed to 2,500 years. For each community we searched for the minimum number of such founding lineages, each with a frequency greater than 5% that would cumulatively account for 40% of the overall mtDNA sample variation. The threshold of 40% was selected to allow comparison to the maternal founding events that we have previously reported [5] for Ashkenazi Jews, among whom 4 founding mothers accounted for 40% of the entire mtDNA genetic variation. The minimum threshold of 5% for inclusion was chosen as it has been previously shown using control-region databases, that frequently derived, phylogenetically recent lineages typically comprise 3%–4% of the total samples for a community [29]. However, it should be kept in mind, that since both the real initial conditions in terms of Hg and lineage composition, and the subsequent evolutionary process that each community underwent from the time of its establishment are unknown, it is obvious that any lineage present in the contemporary population, irrespective of its current frequency, might have been a founder lineage at time the community was established, while an unknown number of founder lineages might have been lost [30]. Therefore, as stated above, our search identifies the list of “successful” founders with respect to the contemporary mtDNA pools of the corresponding Jewish Diaspora communities. One sample from each putative founding lineage, as defined above, was then randomly chosen for complete mtDNA sequencing. Each complete mtDNA was then compared to all published complete mtDNA sequences of its respective Hg to determine “diagnostic” mtDNA sequence positions that would be private or specific to the founding subHg of interest [5], [8], [12], [14]–[16], [18], [22], [24], [31]–[34]. Finally, these putative diagnostic positions from different regions of the mtDNA were genotyped in samples that showed fewer than three control region substitution differences from the respective sample that was completely sequenced (Tables S1 and Table S4).

### Coalescence analysis

The genotyping strategy we followed can determine whether a given founding lineage of interest appears to be monophyletic at a time depth more recent than the time to the most recent common ancestor (TMRCA) of the Hg to which the lineage belongs. However, this does not necessarily address the major research question of the current study, which is to identify the minimal possible number of founders of mtDNA lineages within the approximately 2,000 year historical time frame relevant to the historical context of the Jewish Diaspora that would explain a large proportion of extant mtDNA variation in that population. In other words, we want to assess whether the reduced genetic diversity of mtDNA lineages what can be observed among the Ashkenazi Jews is something particular to their demographic history or common to all Jewish communities derived from the Diaspora. Therefore, it is important to reasonably show that the lineages expanded from single founders within such a time frame, or conversely that a given lineage coalesces within such a time frame, before designating them as founder lineages for the community of interest. To improve the calculation of our coalescence estimates we first expanded the control region sequencing range for dating purposes by calibrating the mutation rate from the estimates derived from complete sequence data. Second, we combined control and partial coding region information known to have different mutation rates. Third, the short time frame of genealogical interest mandates an approach carefully testing the coalescence confidence intervals of any given potential founding lineage, rather than using simple coalescence analysis. Thus, for example, for any lineage based on a sample size less than 10, the simple application of widely accepted mutation rates and SD calculation techniques for HVS-I based coalescence age estimation, will of necessity yield an SD estimate, which is itself greater than the 2,000 year historical bracket of relevance, even if all of the haplotypes are identical [35]–[37]. Fourth, the putative founding lineage might demonstrate star or non star-like phylogeny which affects the coalescence analysis. To overcome these problems we followed a systematic approach to address each of these potential confounding problems.

The mtDNA control region genotyping results in the current study spanned np 16024 to 00300. However, mutational rates for the control region were previously estimated only for a sub-region of the HVS-I, namely, 16093-16383 [36]. To obtain an estimation of the average mutation rates for the control region segment which we used in the current study, we chose the set of Hg K complete mtDNA sequences previously reported [5] and compared the total number of coding region synonymous transition mutations to the total number of transitions and transversions observed in the control region of the same complete mtDNA sequences (Figure S2). The subset of Hg K1 complete mtDNA sequences was analyzed in this manner, and a total of 92 coding region synonymous transitions and 72 control region (16024-00300) transitions and transversions were found. We used the mutation rate from Kivisild et al. [34] of an average rate of 6764 years per synonymous transition in the coding region, corresponding to 8642 years per mutation in the control region segment 16024-00300.

Next, we adapted the ρ statistic of Forster et al. [36] and Saillard et al. [37] to include the sequence and genotyping information obtained from both the control and coding region into the TMRCA calculation as follows. Assume we have n samples from the genomic region under consideration (i.e. the control region), and for m<n samples we also have genotype information for a second region (i.e. the coding region). Assume also that mutation counts have a Poisson distribution, which is exactly true under simple substitution models (Kimura's 3 parameter model or simpler), and approximately true under more complicated models.

The method of Saillard et al. [37] leads to a TMRCA estimate of
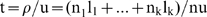
where n_1_ … ,n_k_ are the number of observed samples along the links of the tree, and l_1_ … ,l_k_ are the numbers of mutations in the main (control) region on these links, respectively, and u is the mutation rate for this region (mutations per year).

Assume we also have m_1_,…,m_k_ observed samples of the second genomic (coding) region along the links of the tree, for which we observe r_1_,…,r_k_ mutations, respectively, and that the mutations in the second region occur at a rate of v per year. Then we propose the modified estimator:
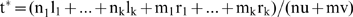
with estimated variance:
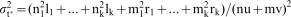



We can prove the following properties of our modified estimator:

Theorem 1

1. Var(t*)<Var(t) whenever 0<m≤2 (i.e., if we observe one or two samples in the second region).

2. If the phylogeny of the sample set is star like, then t* has the lowest variance among unbiased estimators of the lineage TMRCA, which are convex combinations of the unbiased estimators based on the two separate genetic regions.

Proof: Part 1: Assume m = 2, and TMRCA is T, then it follows that:
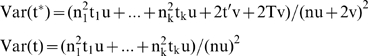
Where t′≤T is the difference between TMRCAs of our two “coding region” samples and the whole lineage.

We now observe a few simple relationships:
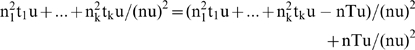
(1)

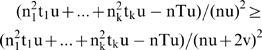
(2)

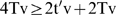
(3)

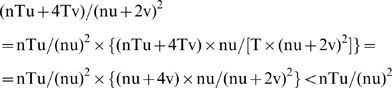
(4)


Putting (1)–(4) together we obtain:
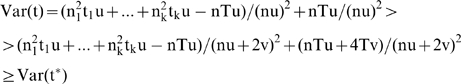



For m = 1 the proof is even simpler, along the same lines.

Part 2: This an immediate result of the Rao-Blackwell theorem [38], since under star phylogeny, the total number of mutations has a Poisson distribution, and for any unbiased estimator t**, which is a linear combination of the two unbiased t estimators from the two regions, it follows that 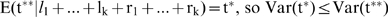


This theorem shows that if we have only one or two coding region samples, then we are always gaining a reduction in variance by combining the information from the two genomic regions, no matter what the true phylogeny is; and that, subject to the star assumption, our estimator optimally combines the estimators calculated from each region separately, irrespective of relative sample size. Under different assumptions, the optimal combination is difficult, often impossible, to calculate.

Finally, to calculate confidence intervals based on TMRCA estimates, we use our estimate σ_t*_^2^ in a normal approximation, as proposed by Saillard et al. [37]. As an alternative approach, we can also observe that if the lineage follows a *star phylogeny* (or more accurately, a *star pedigree*, where all the lines of descent truly coalesce at the MRCA), then n_1_ = … = n_n = _1 and m_1_ = … = m_m_ = 1. In that case, t*×(nu+mv) has a Poisson distribution, and we can use accepted methods for calculating confidence intervals for a Poisson parameter [39]. Here we adopt the confidence interval based on a Pearson approximation. Poisson based calculations are more appropriate than the normal approximation for a star phylogeny. Thus, we employ this Poisson-based inference when the empirical phylogeny we construct for a lineage (based on observed mutations) is consistent with a star phylogeny, and resort to the normal approximation only when the observed data contradicts a star phylogeny.

## Supporting Information

Text S1 Legends for Figures S1 and S2 and Supporting Information References
(0.07 MB DOC)

Figure S1 Phylogeny of all complete mtDNA sequences analyzed in this study
(0.22 MB XLS)

Figure S2 A revised most parsimonious tree of complete Hg K mtDNA
(0.12 MB XLS)

Table S1 Control-region haplotype and haplogroup labeling
(0.32 MB XLS)

Table S2 HVS-I motifs and coding region SNPs used for the initial Hg labeling
(0.03 MB XLS)

Table S3 Haplogroup distribution among the studied populations and communities
(0.12 MB XLS)

Table S4 The analysis of the putative founding lineages
(0.04 MB XLS)

Table S5 Newly introduced nomenclature
(0.03 MB XLS)

## References

[1] DellaPergola S, DellaPergola S, Even S (2001). Jewish Demography 2001.. Papers in Jewish Demography.

[2] Hammer MF, Redd AJ, Wood ET, Bonner MR, Jarjanazi H (2000). Jewish and Middle Eastern non-Jewish populations share a common pool of Y-chromosome biallelic haplotypes.. Proc Natl Acad Sci U S A.

[3] Behar DM, Garrigan D, Kaplan ME, Mobasher Z, Rosengarten D (2004). Contrasting patterns of Y chromosome variation in Ashkenazi Jewish and host non-Jewish European populations.. Hum Genet.

[4] Behar DM, Hammer MF, Garrigan D, Villems R, Bonne-Tamir B (2004). MtDNA evidence for a genetic bottleneck in the early history of the Ashkenazi Jewish population.. Eur J Hum Genet.

[5] Behar DM, Metspalu E, Kivisild T, Achilli A, Hadid Y (2006). The matrilineal ancestry of Ashkenazi Jewry: portrait of a recent founder event.. Am J Hum Genet.

[6] Thomas MG, Weale ME, Jones AL, Richards M, Smith A (2002). Founding mothers of Jewish communities: geographically separated Jewish groups were independently founded by very few female ancestors.. Am J Hum Genet.

[7] Shen P, Lavi T, Kivisild T, Chou V, Sengun D (2004). Reconstruction of patrilineages and matrilineages of Samaritans and other Israeli populations from Y-chromosome and mitochondrial DNA sequence variation.. Hum Mutat.

[8] Reidla M, Kivisild T, Metspalu E, Kaldma K, Tambets K (2003). Origin and diffusion of mtDNA haplogroup X.. Am J Hum Genet.

[9] Richards M, Macaulay V, Hickey E, Vega E, Sykes B (2000). Tracing European founder lineages in the Near Eastern mtDNA pool.. Am J Hum Genet.

[10] Kivisild T, Reidla M, Metspalu E, Rosa A, Brehm A (2004). Ethiopian mitochondrial DNA heritage: tracking gene flow across and around the gate of tears.. Am J Hum Genet.

[11] Chaubey G, Metspalu M, Kivisild T, Villems R (2007). Peopling of South Asia: investigating the caste-tribe continuum in India.. Bioessays.

[12] Roostalu U, Kutuev I, Loogväli EL, Metspalu E, Tambets K (2007). Origin and expansion of haplogroup h, the dominant human mitochondrial DNA lineage in west eurasia: the near eastern and caucasian perspective.. Mol Biol Evol.

[13] Metspalu M, Kivisild T, Metspalu E, Parik J, Hudjashov G (2004). Most of the extant mtDNA boundaries in south and southwest Asia were likely shaped during the initial settlement of Eurasia by anatomically modern humans.. BMC Genet.

[14] Palanichamy MG, Sun C, Agrawal S, Bandelt HJ, Kong QP (2004). Phylogeny of mitochondrial DNA macrohaplogroup N in India, based on complete sequencing: implications for the peopling of South Asia.. Am J Hum Genet.

[15] Sun C, Kong QP, Palanichamy MG, Agrawal S, Bandelt HJ (2006). The dazzling array of basal branches in the mtDNA macrohaplogroup M from India as inferred from complete genomes.. Mol Biol Evol.

[16] Loogväli EL, Roostalu U, Malyarchuk BA, Derenko MV, Kivisild T (2004). Disuniting uniformity: a pied cladistic canvas of mtDNA haplogroup H in Eurasia.. Mol Biol Evol.

[17] Pereira L, Richards M, Goios A, Alonso A, Albarran C (2005). High-resolution mtDNA evidence for the late-glacial resettlement of Europe from an Iberian refugium.. Genome Res.

[18] Achilli A, Rengo C, Magri C, Battaglia V, Olivieri A (2004). The molecular dissection of mtDNA haplogroup H confirms that the Franco-Cantabrian glacial refuge was a major source for the European gene pool.. Am J Hum Genet.

[19] Kivisild T, Rootsi S, Metspalu M, Metspalu E, Parik J, Bellwood P, Renfrew C (2003). The genetics of language and farming spread in India.. Examining the farming/language dispersal hypothesis.

[20] Kivisild T, Kaldma K, Metspalu M, Parik J, Papiha SS, Papiha SS, Deka R, Chakraborty R (1999). The place of the Indian mitochondrial DNA variants in the global network of maternal lineages and the peopling of the Old World.. Genomic diversity: Kluwer Academic/Plenum Publishers.

[21] Mountain JL, Hebert JM, Bhattacharyya S, Underhill PA, Ottolenghi C (1995). Demographic history of India and mtDNA-sequence diversity.. Am J Hum Genet.

[22] Olivieri A, Achilli A, Pala M, Battaglia V, Fornarino S (2006). The mtDNA legacy of the Levantine early Upper Palaeolithic in Africa.. Science.

[23] Forster P (2004). Ice Ages and the mitochondrial DNA chronology of human dispersals: a review.. Philos Trans R Soc Lond B Biol Sci.

[24] Torroni A, Achilli A, Macaulay V, Richards M, Bandelt HJ (2006). Harvesting the fruit of the human mtDNA tree.. Trends Genet.

[25] Torroni A, Bandelt HJ, Macaulay V, Richards M, Cruciani F (2001). A signal, from human mtDNA, of postglacial recolonization in Europe.. Am J Hum Genet.

[26] Andrews RM, Kubacka I, Chinnery PF, Lightowlers RN, Turnbull DM (1999). Reanalysis and revision of the Cambridge reference sequence for human mitochondrial DNA.. Nat Genet.

[27] Richards MB, Macaulay VA, Bandelt H-J, Sykes BC (1998). Phylogeography of mitochondrial DNA in western Europe.. Ann Hum Genet.

[28] Taylor RW, Taylor GA, Durham SE, Turnbull DM (2001). The determination of complete human mitochondrial DNA sequences in single cells: implications for the study of somatic mitochondrial DNA point mutations.. Nucleic Acids Res.

[29] Finnilä S, Lehtonen MS, Majamaa K (2001). Phylogenetic network for European mtDNA.. Am J Hum Genet.

[30] Haak W, Forster P, Bramanti B, Matsumura S, Brandt G (2005). Ancient DNA from the first European farmers in 7500-year-old Neolithic sites.. Science.

[31] Achilli A, Rengo C, Battaglia V, Pala M, Olivieri A (2005). Saami and berbers–an unexpected mitochondrial DNA link.. Am J Hum Genet.

[32] Coble MD, Just RS, O'Callaghan JE, Letmanyi IH, Peterson CT (2004). Single nucleotide polymorphisms over the entire mtDNA genome that increase the power of forensic testing in Caucasians.. Int J Legal Med.

[33] Herrnstadt C, Elson JL, Fahy E, Preston G, Turnbull DM (2002). Reduced-median-network analysis of complete mitochondrial DNA coding-region sequences for the major African, Asian, and European haplogroups.. Am J Hum Genet.

[34] Kivisild T, Shen P, Wall DP, Do B, Sung R (2006). The role of selection in the evolution of human mitochondrial genomes.. Genetics.

[35] Bandelt HJ, Forster P, Röhl A (1999). Median-joining networks for inferring intraspecific phylogenies.. Mol Biol Evol.

[36] Forster P, Harding R, Torroni A, Bandelt HJ (1996). Origin and evolution of Native American mtDNA variation: a reappraisal.. Am J Hum Genet.

[37] Saillard J, Forster P, Lynnerup N, Bandelt HJ, Norby S (2000). mtDNA variation among Greenland Eskimos: the edge of the Beringian expansion.. Am J Hum Genet.

[38] Casella G, Berger R (2001). Statistical Inference: Duxbury Press..

[39] Johnson NI, Kotz S (1969). Discrete Distributions..

