# Supplementary material for: Counting the Founders: The Matrilineal Genetic Ancestry of the Jewish Diaspora
Source: PLoS One. 2008 Apr 30;3(4):e2062. doi: 10.1371/journal.pone.0002062 (PMC2323359; doi:10.1371/journal.pone.0002062)
Supplement: Text S1 — Legends for Figures S1 and S2 and Supporting Information References (0.07 MB DOC) [file pone.0002062.s001.doc]

**Supplemental Figures legends**

Figure S1

A phylogenetic tree comprised of the 49 complete mtDNA sequences analyzed in this study and 4 additional complete mtDNA sequences frequent among Ashkenazi Jews as previously reported by Behar et al. [1]. The tree was composed to facilitate the reading of the paper and to aid in the assignment of the newly reported haplotypes. We included in the tree all previously reported clades of the human mtDNA phylogeny in which our founding lineages were found. For the specific case studies of the Georgian *HV1a1a1*, Azerbaijani *J2b1* and Lybian X2e1a1a lineages, we superimposed on the tree the information that became available to us from the extended phylogenetic analysis. Therefore, the tree is rooted following Kivisild et al. [2] and is not most parsimonious for this limited dataset, as many major branching positions rely on prior information reported in the literature and as well on our unpublished complete mtDNA sequences, and not solely on the novel data presented herein [1-14]. Mutations are shown on the branches and are transitions, unless the base change is explicitly indicated in lower case letters. Deletions are indicated by a “d” following the deleted nucleotide position. Insertions are indicated by a dot followed by number and type of inserted nucleotide(s). The ampersand sign (@) at the end of a nucleotide position(s) denotes a reversion to the ancestral state in the relative pathway to the rCRS. The information of the reported samples is presented in Table 2, Table S4 and Table S5. Nucleotide positions in red correspond to the diagnostic positions inferred from the complete sequence information and checked in all samples thought to represent a monophyletic lineage based on the control region information. The tree was drawn by hand and the indels at nucleotide positions 309 and 315 were excluded.

While drawing the tree we did not change any label designations already in the current literature. Therefore, the polymorphisms labeled on each of the internal links follow exactly their location in the citation from which they have been retrieved. The polymorphisms shown on the links connecting the new haplotypes represent all novel polymorphisms recorded herein. However, it is common practice to label ancestral and descendant clades that are linked by a few positions without reserving labels to a possible intermediate branch, as in the case of J1 and J1b that are separated by six mutational events [10]. In a few cases, such as the noted J1-J1b, our data revealed an intermediary branch, causing a labeling dilemma. In this specific case, we draw the bifurcation and named the novel sister clade as J1e and the bifurcation as J1b’e. In that sense, the string of positions from J1 to J1b remains the same but are now divided to be in the pre or post bifurcation links. We also faced a problem while trying to label our novel haplotypes, as we examined only a subset of possible coding region positions for each founding lineage. This limits the ability to accurately define its complete internal sub-clade structure (Table S5). In theory, any position not genotyped could have been at the root of the newly suggested clade or private to the very specific sequenced mtDNA genome. We designated the positions genotyped in all samples belonging to the founding lineage in the link leading to the new labeled segregating site, and all the other in the following link.. We used italic fonts to label these haplotypes, with the understanding that these labels are given only to facilitate reading, and as prospective candidates of clades to be fully defined with future comprehensive genotyping where relevant (Table S5).

Figure S2

A revised most parsimonious tree of complete Hg K mtDNA sequenced previously, as reported by Behar et al. [1]. The tree differs from the previously reported topology map as follows: 1) A few typographic errors were identified and corrected as shown in blue. 2) A few mistakes that were the result of using the original posted data by Herrnstadt et al. [7] and not the later erratum correction by Herrnstadt et al. [15] (we thank Hans Bandelt for bringing this to our attention). It is important to note that the transition C7927G previously identified as a phantom mutation is confirmed herein as real. 3) The indel positions at nucleotide position 523 are now included. 4) Transition and transversions are both labeled in upper and lower case, respectively. 5) Information regarding amino acid replacements are specified to the side of the noted position; synonymous are labeled by (s), non-synonymous by (ns), non-coding by (nc), change in tRNA gene by (t) and change in rRNA gene by (r).

**Supplementary Information References**

1. Behar DM, Metspalu E, Kivisild T, Achilli A, Hadid Y, et al. (2006) The matrilineal ancestry of Ashkenazi Jewry: portrait of a recent founder event. Am J Hum Genet 78: 487-497.

2. Kivisild T, Shen P, Wall DP, Do B, Sung R, et al. (2006) The role of selection in the evolution of human mitochondrial genomes. Genetics 172: 373-387.

3. Achilli A, Rengo C, Magri C, Battaglia V, Olivieri A, et al. (2004) The molecular dissection of mtDNA haplogroup H confirms that the Franco-Cantabrian glacial refuge was a major source for the European gene pool. Am J Hum Genet 75: 910-918.

4. Behar DM, Villems R, Soodyall H, Blue-Smith J, Pereira L, et al. (2007) The dawn of human matrilineal diversity: Insights from complete mitochondrial genomes. Submitted.

5. Achilli A, Rengo C, Battaglia V, Pala M, Olivieri A, et al. (2005) Saami and berbers--an unexpected mitochondrial DNA link. Am J Hum Genet 76: 883-886.

6. Coble MD, Just RS, O'Callaghan JE, Letmanyi IH, Peterson CT, et al. (2004) Single nucleotide polymorphisms over the entire mtDNA genome that increase the power of forensic testing in Caucasians. Int J Legal Med 118: 137-146.

7. Herrnstadt C, Elson JL, Fahy E, Preston G, Turnbull DM, et al. (2002) Reduced-median-network analysis of complete mitochondrial DNA coding-region sequences for the major African, Asian, and European haplogroups. Am J Hum Genet 70: 1152-1171.

8. Loogvali EL, Roostalu U, Malyarchuk BA, Derenko MV, Kivisild T, et al. (2004) Disuniting uniformity: a pied cladistic canvas of mtDNA haplogroup H in Eurasia. Mol Biol Evol 21: 2012-2021.

9. Olivieri A, Achilli A, Pala M, Battaglia V, Fornarino S, et al. (2006) The mtDNA legacy of the Levantine early Upper Palaeolithic in Africa. Science 314: 1767-1770.

10. Palanichamy MG, Sun C, Agrawal S, Bandelt HJ, Kong QP, et al. (2004) Phylogeny of mitochondrial DNA macrohaplogroup N in India, based on complete sequencing: implications for the peopling of South Asia. Am J Hum Genet 75: 966-978.

11. Reidla M, Kivisild T, Metspalu E, Kaldma K, Tambets K, et al. (2003) Origin and diffusion of mtDNA haplogroup X. Am J Hum Genet 73: 1178-1190.

12. Roostalu U, Kutuev I, Loogvali EL, Metspalu E, Tambets K, et al. (2007) Origin and expansion of haplogroup h, the dominant human mitochondrial DNA lineage in west eurasia: the near eastern and caucasian perspective. Mol Biol Evol 24: 436-448.

13. Sun C, Kong QP, Palanichamy MG, Agrawal S, Bandelt HJ, et al. (2006) The dazzling array of basal branches in the mtDNA macrohaplogroup M from India as inferred from complete genomes. Mol Biol Evol 23: 683-690.

14. Torroni A, Achilli A, Macaulay V, Richards M, Bandelt HJ (2006) Harvesting the fruit of the human mtDNA tree. Trends Genet 22: 339-345.

15. Herrnstadt C, Preston G, Howell N (2003) Errors, phantoms and otherwise, in human mtDNA sequences. Am J Hum Genet 72: 1585-1586.
